# Thermal-Flow Characteristics of Ferrofluids in a Rotating Eccentric Cylinder under External Magnetic Force

**DOI:** 10.3390/mi9090457

**Published:** 2018-09-12

**Authors:** Jae-Hee Kim, Hyeon-Seok Seo, Youn-Jea Kim

**Affiliations:** 1 Graduate School of Mechanical Engineering, Sungkyunkwan University, Suwon 16419, Korea; jaehee29@skku.edu; 2 Chassis-Trim Analysis Team, Hyundai Mobis, Yongin 16891, Korea; bbashya00@gmail.com; 3 School of Mechanical Engineering, Sungkyunkwan University, Suwon 16419, Korea

**Keywords:** ferrofluid, magnetic body force, computational fluid dynamics (CFD), finite element method (FEM), magnetic material

## Abstract

Heat dissipation has become an important issue due to the miniaturization of various electronic devices. Various methods such as spray and nozzle coolers, heat sinks and so on are used for heat dissipation. However, the emergence of ferrofluids drastically improves the operating characteristics of electromagnetic systems and devices. A ferrofluid is a suspension containing 10-nm magnetic particles in a colloidal solution. This material exhibits paramagnetic behavior and is sensitive to magnetic field and temperature. In this study, heat transfer characteristics of ferrofluids in a rotating eccentric cylinder were investigated using the commercial code, COMSOL Multiphysics. Numerical results of the local Nusselt number, magnetophoretic force and velocity distributions were obtained from various eccentricities of the cylinder, and the results were graphically depicted with various flow conditions.

## 1. Introduction

Due to the miniaturization of electronic devices, heat dissipation has become an essential problem to be solved. In particular, the motor generates heat due to various causes such as ambient temperature, dust and obstacles. If the heat is not released quickly, the performance of the motor may deteriorate. In addition, if the permanent magnet of the motor is exposed to a high-temperature environment or has insufficient heat release performance, it loses its inherent characteristics and its performance drops sharply and may directly affect the efficiency of the motor [1]. Convective heat transfer inside concentric and eccentric rings has many applications in scientific and engineering fields such as electric motors and generators, completion of oil sources, and heating and cooling of underground electrical cables [2]. Matin and Pop [2] also presented a numerical study of the natural convective heat transfer of Cu–water nanofluids in eccentric horizontal rings. The governing equations were derived from the polar coordinate system for the eccentric physical domain and then transformed into the rectangular region, and showed the effect of eccentricity, radius ratio, nanoparticle volume ratio, Rayleigh number and Prandtl number on the flow and heat transfer. Recent studies on the effect of eccentricity on heat transfer have been of interest to most researchers, and research for dissipating the heat inside the motor by various methods is continuously performed. Jeng [3] attempted to dissipate the heat of the motor using a finned heat sink and a motor fan, and achieved about 28% higher heat dissipation than the previous model. Li et al. [4] improved the heat dissipation structure for effective heat management, and optimized the ventilation channel size to obtain high heat dissipation effect. In addition to modifying the structural characteristics, it is possible to obtain a heat dissipation effect by removing the air layer that serves as an insulator by filling the air layer inside the motor with insulating oil [5]. Recently, a variety of fluids for heat dissipation have been used by using this method, and ferrofluids, which are known to have good heat dissipation performance, are mainly used. The ferrofluid is a colloidal solution with magnetic particles of 10-nm size, and it behaves like a general fluid when a magnetic field is not applied. However, a non-Newtonian motion is performed when a magnetic field is applied. In addition, the thermal conductivity is about 5.7-times better than that of air, and at the same time it has a sealing effect [6].

Petit et al. [7] experimentally confirmed the production of static pressure using thermomagnetic coupling when applying a magnetic field using a ferrofluid flowing by nonmechanical effects. Kim et al. [8] experimentally confirmed the heat flow characteristics and the changes of the external magnetic field in the concentric circular tube. The effect of the applied magnetic field on the diameter of the double tube affects the heat flow characteristics. Yang et al. [9] conducted a numerical study on the natural convection of eccentric annulus, and found that the fluid containing nanoparticles easily changed the flow pattern and improved heat transfer by about 16% compared to pure fluids. Abu-Nada et al. [10] confirmed the enhancement of the heat transfer of ferrofluids in concentric circular tubes using aqueous ferrofluids. As the ratio of the inner circular tube to the gap value increases, the presence of the particles changes and heat transfer improves. Sheikholeslami et al. [11] studied the effect of magnetohydrodynamics (MHD) on the free convection of nanofluids in eccentric half-rings. The lattice Boltzmann method was used and the results show that the Nusselt number is a direct function of the volume fraction of the nanoparticles and the Rayleigh number, and has an inverse correlation with the Hartmann number and the position of the inner cylinder.

Studies on heat transfer using ferrofluids in various forms and fields are underway. However, there are few studies on the heat transfer characteristics in the eccentric circular cylinder when the rotation is applied. Therefore, in this study, the heat dissipation performance was confirmed by using the ferrofluid filled between the rotating eccentric cylinder and outer case. In particular, heat dissipation performance was confirmed by the degree of eccentricity of the internal rotation model.

## 2. Methodology

**Figure 1:**
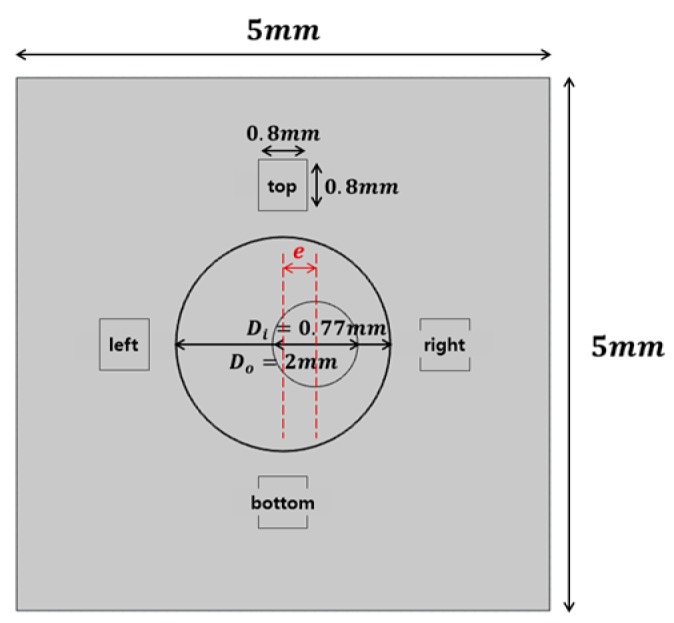
Schematic of the modeled channel.

A two-dimensional microchannel was created to clearly compare the degree of eccentricity and rotation of the motor. The microchannel has an annular shape composed of a motor and a static outer tube rotating inward in a sealed state, as shown in Figure 1, and magnets are positioned on each of the top, bottom, right and left sides [12]. In order to confirm the heat dissipation performance, the internal space was filled with ferrofluid, and the magnetic field was located outside the channel. In the reference model, the outer tube and the inner cylinder are concentric and the inner motor is adjusted to increase the eccentricity by 0.1 to the right direction (see Table 1). The working fluid used in this numerical analysis is an oil-based ferrofluid and is the EFH-1 model of Ferrotec. The detailed characteristics are shown in Table 2. A commercially available program, COMSOL Multiphysics, was used to verify the thermal flow of the ferrofluid by applying a magnetic field.

The continuity, momentum and energy equations are used in two-dimensional models to check the flow of the ferrofluid and heat transfer phenomena according to application of the external magnetic field and the rotation of the motor. The governing equations used in this study are as follows [13]:

continuity:

(1) ∇·V=0 ,

momentum:

(2) $$\rho \left(\frac{\partial V}{\partial t}+V\cdot \nabla V\right)=-\nabla p+\mu \nabla^{2}V+\mu_{0}\left(M\cdot \nabla \right)H,$$

energy:

(3) $$\rho C_{p}\left(\frac{\partial T}{\partial t}+V\cdot \nabla T\right)=k\nabla^{2}T-\mu_{0}T\frac{\partial M}{\partial T}\left(\left(V\cdot \nabla \right)H\right),$$

where ρ is the density of the magnetic fluid, μ is the dynamic viscosity, $\mu_{0}$ is the vacuum magnetic permeability, *M* is the magnetization of the ferrofluid and *H* is the magnetic field strength. The last term of the momentum equation represents the Kelvin force per unit volume produced when a ferroelectric is applied to a non-uniform magnetic field.

In the case of small temperature changes, the magnetization of the working fluid can be expressed in the following linear form:

(4) $$M=M^{*}+{\left(\frac{\partial M}{\partial T}\right)}_{H}\left(T-T^{*}\right)+{\left(\frac{\partial M}{\partial H}\right)}_{T}\left(H-H^{*}\right).$$

The magnetization vector can be expressed using the magnetic field vector and the magnetic susceptibility. Considering the case where the susceptibility is estimated only as a function of temperature and the temperature variation is small, the linear relationship for magnetic susceptibility can be expressed as follows:

(5) $$\chi_{m}=\frac{\chi_{0}}{1+\beta \left(T-T^{*}\right)}.$$

**Figure 2:**
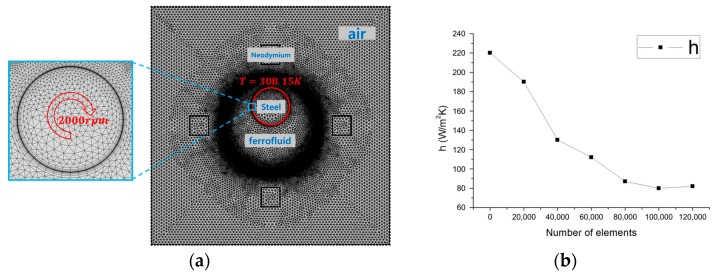
Schematic of the boundary conditions. (**a**) Grid systems; (**b**) Grid dependency test.

A frozen rotor was used to implement a rotating motor. The exothermic temperature was assumed to be 308.15 K. All temperatures except motor temperature are based on room temperature. Detailed boundary conditions are shown in Table 3 and Figure 2a. A free triangular grid element was applied to the whole model and inflation was set to 10 layers in order to clearly confirm the effect of magnetic convection and rotation. In addition, to confirm the suitability of the mesh, the grid dependency test was performed by increasing the total grids from 10,000 to 100,000 uniformly. As a result, it was found that the heat transfer coefficient was converged when there were more than 80,000 grids (see Figure 2b). Therefore, the number of grids for the whole model is set to about 80,000. In order to confirm the validity of the numerical analysis, the experimental results of Kuehn and Goldstein [14] were compared with numerical values and it was confirmed that similar heat transfer flow occurred.

## 3. Results and Discussion

### 3.1. Magnetophoretic Force

The magnetophoretic (*MAP*) force represents the behavior of a ferrofluid due to the interaction of the ferrofluid with a magnetic field gradient, and its value can be expressed as follows:

(6) $${\vec{F}}_{MAP}=\left(\mu_{m}m_{eff}\cdot \nabla \right)\vec{H},$$

and

(7) $${\vec{F}}_{MAP}=2\pi r_{p}^{3}\mu_{m}ReK\left(\mu_{p},\mu_{m}\right)\nabla H^{2}.$$

**Figure 3:**
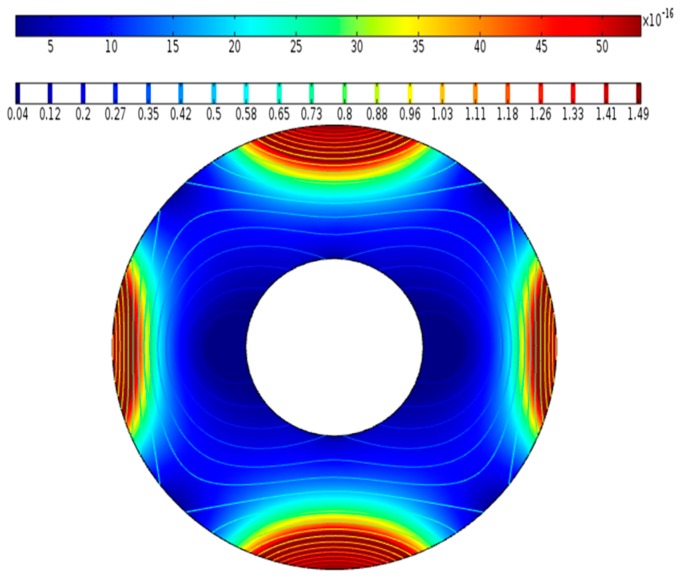
The magnetophoretic (*MAP*) force and magnetization distributions in the reference model.

As shown in Figure 3, the distribution of the *MAP* force for the case of the reference model is similar to the magnetization distribution. This is because the slope of the magnetic field calculated by the external magnetic field is reflected in the *MAP* force distribution given in the above equation. Further, as shown in Figure 3, the magnetic force becomes stronger as the magnetic field is closer to the outside. From these results, we can conclude that the flow changes due to the concentration of uneven *MAP* forces.

### 3.2. Velocity Distribution

**Figure 4:**
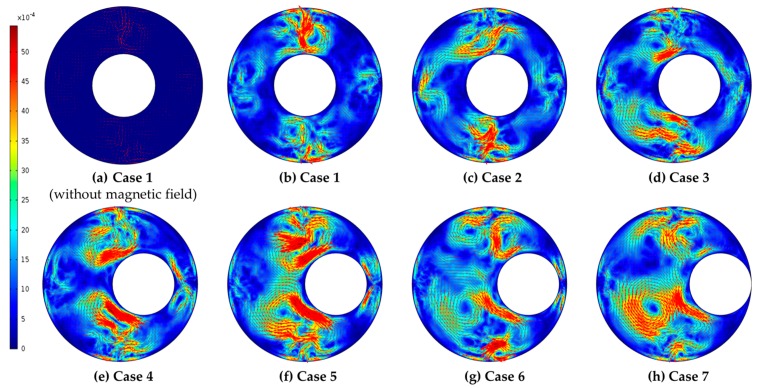
Velocity distributions with various eccentricity conditions for the case of *H* = 10 kA/mm.

When a magnetic field is applied, a vortex field occurs due to an increase or decrease in the *MAP* force. This force is accelerated by the effect of the rotating motor. Therefore, it was confirmed that the vortex field and velocity field distribution, which occur when an external magnetic field is applied, affect the heat transfer. Figure 4 shows the effect of the magnetic field on the model as a velocity field. In Figure 4a, since there is no external magnetic field, only the influence of the inner rotating cylinder is shown, and the speed is low. However, in Figure 4b, an external magnetic field is applied and the ferrofluid flow is activated. It also shows that the center of the magnetic field is fast due to the concentration of the *MAP* force. Figure 4 shows the velocity field for the entire case. When the eccentricity changes, the flow velocity is influenced. Especially in Case 5, the velocity increases in the entire flow region. If the eccentricity is less than 0.3, sufficient flow to the right cannot be activated. If the eccentricity is more than 0.5, it is too close to the outer tube and the flow does not occur easily. Therefore, it was confirmed that when the eccentricity is *e* = 0.4, the largest eddy field and smooth flow occur.

### 3.3. Nusselt Number

To determine the heat transfer of a microchannel in a dimensionless number, check the distribution of the number of local Nusselt numbers and use the following formula to determine the number:

(8) $$Nu\left(x\right)=\frac{h\left(x\right)D}{k_{f}}=\frac{L}{\left(T_{h}-T_{c}\right)}{\left(\frac{\partial T}{\partial y}\right)}_{y=0},$$

where *h* is the heat transfer coefficient, *k* is the thermal conductivity of the ferrofluid, *L* is the length of the channel, and $T_{h},\;T_{c}$ are the high and low temperatures, respectively.

**Figure 5:**
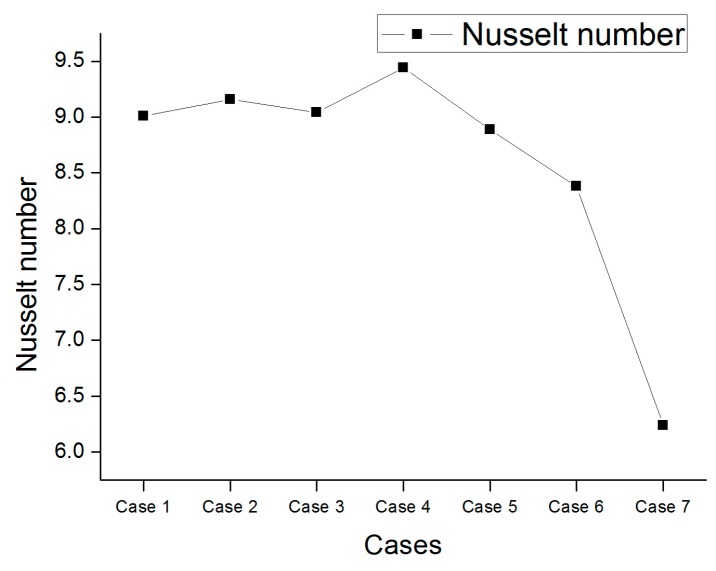
Nusselt number at each case.

**Figure 6:**
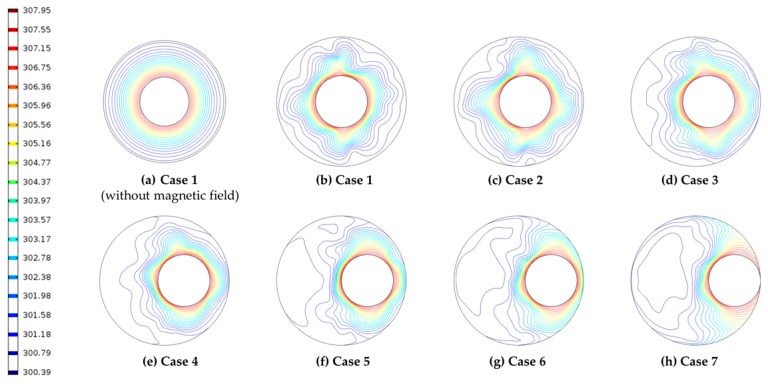
Isotherms at each case.

The Nusselt number is a measure of the convection heat transfer leading to a change in heat transfer, and the heat transfer rate can be determined by the slope. By checking the Nusselt number in each microchannel model, one can control the heat transfer between the surfaces depending on the degree of eccentricity. In addition, thermodynamic convection near the outer magnet has been shown to promote heat transfer. As a result of the numerical analysis, the Nusselt number according to the degree of eccentricity is shown in Figure 5. When more vortices are formed, the average Nusselt number is higher than when the vortex field is not formed. Case 4 shows that the Nusselt number changes rapidly and the heat transfer is higher than the others. This result shows that the eccentricity of the internal motor improves the heat transfer performance by causing a sudden change in the Nusselt number (see Figure 6).

## 4. Conclusions

In this study, the thermal-flow characteristics of ferrofluids in a rotating eccentric cylinder were investigated numerically for seven different cases. The behaviors of the flow and energy transfer of the ferrofluid in the microchannel are obtained as follows:

1. When a magnetic field is applied to a ferrofluid through the permanent magnet, magnetization distribution appears due to magnetic flux density distribution. It was confirmed that the *MAP* force was concentrated in the portion where the magnetic flux density gradient was large. It has also been confirmed that various velocity changes and vortex fields are generated by the *MAP* force.
2. Since the center of the inner cylinder moves away from the center of the outer cylinder, it has a higher Nusselt number because it forms a sufficient flow. However, it has been confirmed that the effect of heat transfer is reduced by direct contact when it is too close to the outer wall. Therefore, the highest Nusselt number was confirmed in Case 4 (*e* = 0.3), which is considered to be the most appropriate location.
3. The eccentricity of the inner cylinder was found to influence the vortex field under the magnetic field. It is considered that a highly efficient heat dissipation system can be constructed by using the characteristic that a change in efficiency is caused by a small eccentricity change. However, a comparative study of the results obtained from the experiment should be performed to confirm the accuracy of numerical studies.
4. The characteristics of the heat transfer according to the eccentricity of the ferrofluids can be applied to various rotating devices such as electric motors and generators.

## Figures and Tables

**Table 1:** Eccentricity of the inner cylinder.

Cases	Eccentricity, *e*
Case 1 (ref.)	0
Case 2	0.1
Case 3	0.2
Case 4	0.3
Case 5	0.4
Case 6	0.5
Case 7	0.6

**Table 2:** Physical properties of the working fluid (ferrofluid).

Properties	Value
ρ	1221 (kg/m^3^)
$\mu_{r}$	2.552
$\varepsilon_{r}$	2.208
$\chi_{m}$	1.552
η	0.00727 (Pa∙s)
*k*	0.19 (W/m∙K)
$C_{p}$	1840 (J/kg∙K)
β	8.6 × 10^−4^ (1/K)

**Table 3:** Boundary conditions applied in this study.

*H*	10 (kA/mm)
$T_{h}$	308.15 (K)
$T_{amb}$	298.15 (K)
Wall condition	No-slip
